# *Terminalia fagifolia* Mart. & Zucc. elicits vasorelaxation of rat thoracic aorta through nitric oxide and K^+^ channels dependent mechanism

**DOI:** 10.1242/bio.035238

**Published:** 2019-01-25

**Authors:** Emanuella F. de Carvalho, André F. Nunes, Náiguel C. B. Silva, João Paulo da Silva Gomes, Renato P. de Sousa, Valdelânia G. Silva, Paulo H. M. Nunes, Rosimeire F. Santos, Mariana H. Chaves, Aldeidia P. Oliveira, Rita C. M. Oliveira

**Affiliations:** 1 Medicinal Plants Research Center, Federal University of Piauí, 64049-550 Teresina, PI, Brazil; 2 Department of Chemistry, Federal University of Piauí, 64049-550 Teresina, PI, Brazil; 3 Department of Biophysics and Physiology, Federal University of Piauí, 64049-550 Teresina, PI, Brazil

**Keywords:** *Terminalia fagifolia*, Vasorelaxation, Potassium channels, Nitric oxide

## Abstract

*Terminalia fagifolia* Mart. & Zucc. (Combretaceae) is a plant commonly found in the regions of the Brazilian cerrado, popularly used for the treatment of gastrointestinal disorders. There are no reports in the literature on the use of *T. fagifolia* for the treatment of the cardiovascular system conditions. Nevertheless, plants of the same genus, such as *Terminalia* *arjuna* (Roxb.) Wight & Arn and *Terminalia* *superba* Engler & Diels, present cardioprotective, hypotensive and vasodilatating effects. In light of this, the aim of the study was to investigate the effect of the ethanolic extract (Tf-EE) and of its aqueous (Tf-AQF), hexanic (Tf-HEXF) and hydroethanolic (Tf-HAF) partition fractions obtained from the stem bark of *T.* *fagifolia* Mart. & Zucc. The effects of the extract and partition fractions of *T. fagifolia* were evaluated on isometric tensions in the thoracic aorta rings of Wistar rats (250–300 g). Tf-EE, Tf-HEXF and Tf-HAF presented a concentration-dependent vasorelaxant effect, and Tf-AQF presented a vasorelaxant effect that was more potent in the presence of endothelium. The relaxation curves of the aorta promoted by the fraction investigated were attenuated in the presence of the following pharmacological tools: L-NAME, ODQ or PTIO. The vasorelaxant effect of the aorta promoted by Tf-AQF was attenuated in the presence of TEA and 4-AP. Tf-EE induced a concentration-dependent and endothelium-independent vasorelaxation. Tf-HAF and Tf-HEXF presented concentration-dependent and vascular-endothelium-independent vasorelaxation, but did not obtain 100% of relaxation. On the other hand, Tf-AQF presented concentration-dependent vasorelaxation that was more potent in aorta rings with vascular endothelium. The relaxant mechanism induced by the Tf-AQF involves the NO/sGC/cGMP pathway and channels Kv.

## Abstract (precis)

**Summary:** The investigation of the relaxing effect of extract and fractions of the stem bark partition of *Terminalia fagifolia* on aortic rings is a pioneering study involving the participation of K^+^ channels, which demonstrates a potential alternative therapeutic method for the treatment of cardiovascular diseases.

## INTRODUCTION

*Terminalia fagifolia* Mart. & Zucc is part of the Combretaceae family, which is composed of by around 600 species distributed in 18 genera. *Terminalia* is composed of by around 200 species. It is a species found in the Brazilian cerrado, popularly-known as capitão-do-mato, mirindiba and pau-de-bicho (Almeida et al., 1998; Ayres et al., 2009).

Several plants of the Combretaceae botanical family present distinct biological properties (Wen et al., 2011). In the *Terminalia* genus, pentacyclic triterpenes are found, as well as their glycosylated derivatives, flavonoids, tannins and other aromatic compounds (Araújo and Chaves, 2005). The thin-layer chromatography of the ethanolic extract of the *T. fagifolia* stem bark suggested the presence of polar compounds, such as flavonoids, glycosylated flavonoids and saponins (Nunes et al., 2014). In popular medicine, the stem bark is used in treatment against aphtha and tumors (Ayres et al., 2009). Besides this, it is used as a digestive aid in the treatment of stomach and intestine conditions (Freire et al., 1992). Its antioxidant, antidiarrheal and gastroprotective activities have already been shown in rodents (Nunes et al., 2014), as well as an antibacterial use for certain sepas (de Araujo et al., 2015). Also, plants of the same species presented vasodilatating activities and also activities protective of the endothelial dysfunctions (Kapoor et al., 2014).

Recent investigations suggest that the *T. fagifolia* extract can exert a number of medicinal effects due to its antioxidant property (Nunes et al., 2014) and a potential antitumor activity (de Araujo et al., 2015). The presence of flavonoids in its constitution may favor the vasorelaxant effect. However, the mechanism of action through which the flavonoids can promote the vasorelaxation has not yet been elucidated (Zhang et al., 2010).

The pathologies that affect the cardiovascular system are the main cause of death in both developed and developing countries (Rosamond et al., 2008), causing a great impact not only to man's health, but also to social and economic areas. In an attempt to reduce this impact, researchers worldwide have worked diligently to improve the treatment of cardiovascular diseases, including the discovery of new therapeutic strategies (Lui et al., 2013).

The vascular endothelium plays a fundamental role in the regulation of the vascular tonus (Villar et al., 2006; Wong et al., 2017) inducing vasorelaxation through the synthesis and release of derivatives of endothelial-relaxing factors, including nitric oxide (NO) and prostacyclin (PGI_2_) (Furchgott and Zawadzki, 1980). The K^+^ channels also play an important role in the regulation of the vascular tonus. As an effect, the K^+^ channels in blood vessels indirectly influence vascular tension, altering the potential of the resting membrane (Qu et al., 2014). Several natural substances can lead to the vasodilatation or vasoconstriction effects, either opening or closing the K^+^ channels (Brereton et al., 2013). The aim of the present study was investigate the vascular effect of the ethanolic extract of *T. fagifolia* and of its aqueous, hexanic and hydroethanolic partition fractions in the thoracic aorta isolated from rats, and elucidate the mechanism that is subjacent to this vascular activity.

## RESULTS

### FTIR and UV-Vis fingerprinting of extracts and fractions

FTIR and UV-Vis fingerprinting of the extract and fractions of *T. fagifolia* are shown in Fig. S1 and Fig. S2. The analysis of these spectra suggested the presence of phenolic compounds (flavonol) in Tf-EE, Tf-HAF and Tf-AQF, as well as lipidic nature (Tf-EE and Tf-HEXF).

### Effects of Tf-EE and its partition fraction on the contraction of rat thoracic aortic rings with intact or denuded endothelium induced by PHE

**Fig. 1.**
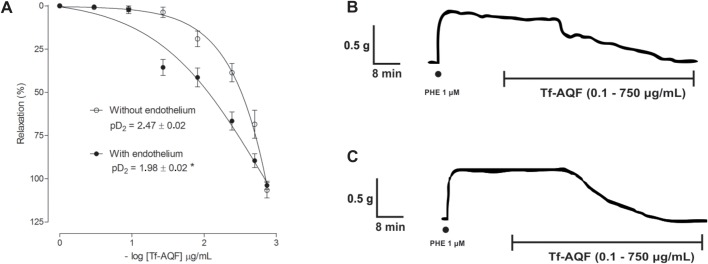
**Effect of Tf-AQF on PHE-induced (1 µM) tonic contraction in rat aorta rings with (E+) or without (E−) endothelium.** Symbols and vertical bars represent means and s.e.m., *t*-test, *P<0.05 (with endothelium versus without endothelium) (*n*=5) (A), and original records (B,C).

Cumulative addition of Tf-EE, Tf-HEXF, Tf-HAF or Tf-AQF (0.1–750 μg/ml) in thoracic aortic rings pre-contracted by PHE presented a concentration-dependent vasorelaxant effect with E_max_ values of at least 100% (Table 1). The effect produced by Tf-AQF, besides being effective above 100%, induced vasorelaxation with greater potency in the presence of the endothelium (Table 1 and Fig. 1).

**Table 1.**
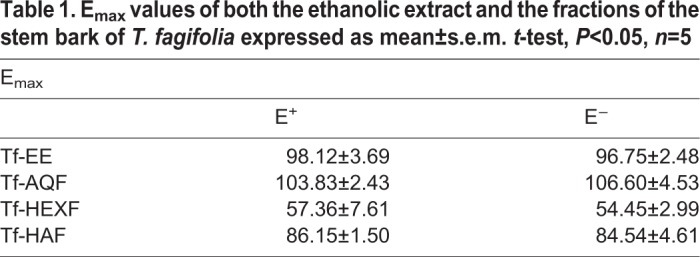
**E_max_ values of both the ethanolic extract and the fractions of the stem bark of *T. fagifolia* expressed as mean±s.e.m. *t*-test, *P*<0.05, *n*=5**

### Role of endothelium in Tf-AQF-induced relaxation

**Fig. 2.**
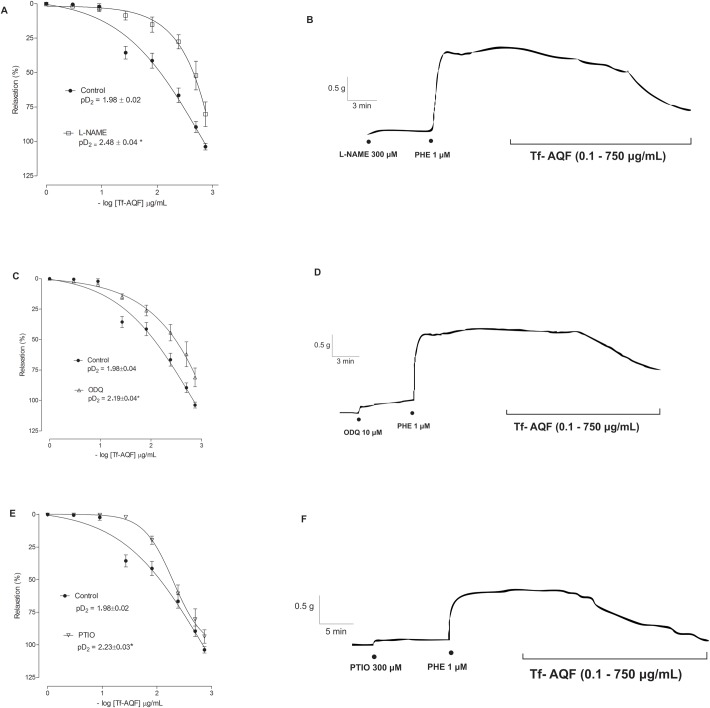
**Effect of Tf-AQF on PHE-induced (1 μM) tonic contraction in rat aorta rings with endothelium in the absence (control) and presence of the pharmacological tool L-NAME (300 μM, *n*=5) (A,B), ODQ (10 μM, *n*=4) (C,D) or PTIO (300 μM, *n*=5) (E,F).** Symbols and vertical bars represent means and s.e.m., *t*-test, *P<0.05 (control versus pharmacological tool).

In order to evaluate the involvement of endothelium-derived relaxing factors in Tf-AQF-induced vasorelaxation, L-NAME (300 μM), an inhibitor of eNOS, ODQ (10 μM), a soluble guanylyl cyclase inhibitor and PTIO (300 μM), a free radical scavenger, were used in the presence of the extract. Because the NO-cGMP signaling is important in the regulation of endothelium-dependent vasorelaxation, effects of an inhibition from endothelial NO synthase (eNOS), soluble guanylyl cyclase (sGC) and nitric oxide (NO) activity was examined. As shown in Fig. 2A–F, the pretreatment in aortic rings with L-NAME, ODQ or PTIO significantly attenuated Tf-AQF-induced vasorelaxation. These findings suggest that an activation of NO/sGC/cGMP signaling is involved in Tf-AQF-induced vasorelaxation.

### Role of K^+^ channel in Tf-AQF-induced relaxation

**Fig. 3.**
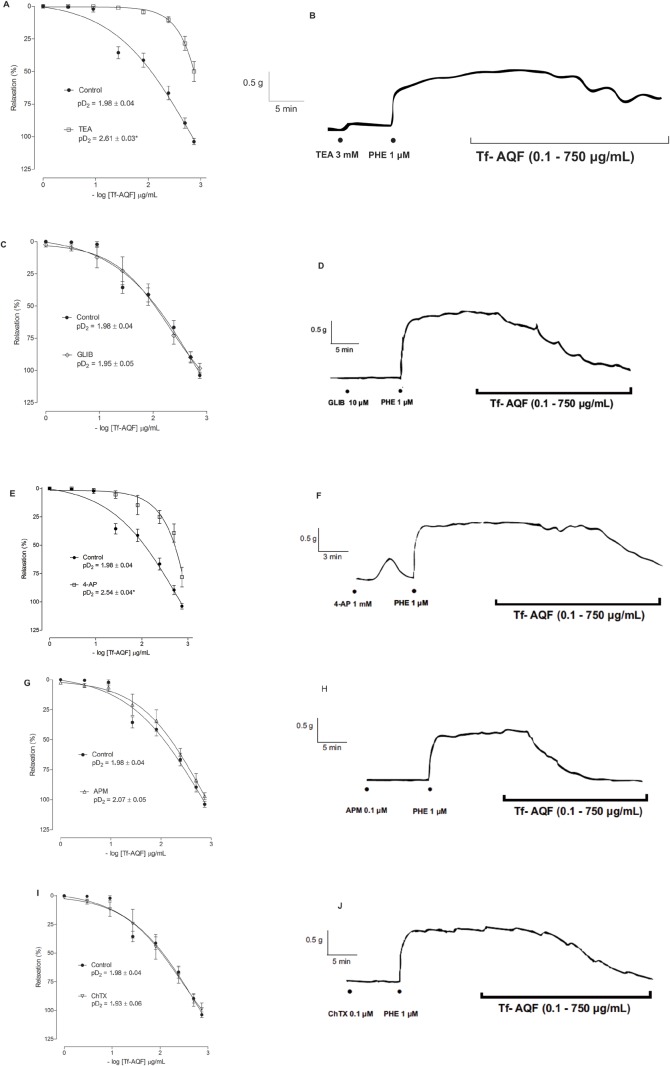
**Effect of Tf-AQF on PHE-induced (1 μM) tonic contraction in rat aorta rings with endothelium in the absence (control) and presence of the pharmacological tool TEA (3 mM, *n*=5) (A,B), GLIB (10 μM, *n*=5) (C,D), 4-AP (1 mM, *n*=4) (E,F), APM (1 μM, *n*=3) (G,H) and ChTX (0.1 μM, *n*=3) (I,J).** Symbols and vertical bars represent means and s.e.m., *t*-test, *P<0.05 (control versus pharmacological tool).

In order to test the involvement of the K^+^ channels in Tf-AQF-induced vasorelaxation, the effects of many K^+^ channel blockers were evaluated. As shown in Fig. 3A,B, the Tf-AQF-induced vasorelaxation was attenuated by a non-selective blocker of K^+^ channels, TEA (3 mM); the pretreatment with an ATP-sensitive K^+^ channels (K_ATP_) blocker, GLIB (10 μM), had no significant effect on the relaxation (Fig. 3C,D). The relaxation induced by Tf-AQF on the endothelium-intact aortic rings pre-contracted with PHE was significantly attenuated in the presence of 4-AP (1 mM), a voltage-dependent K^+^ channel (K_V_) blocker (Fig. 3E,F). To further define the role of Ca^2+^-activated K^+^ channels (K_Ca_) in Tf-AQF-induced vasorelaxation, the effects of K_Ca_ channel blockers were tested. As shown in Fig. 3G,H, Tf-AQF-induced vasorelaxation was attenuated by a selective blocker of small-conductance K_Ca_ (SK_Ca_) APM (1 μM) or a selective blocker of intermediate-conductance K_Ca_ channel (IK_Ca_). ChTX (0.1 μM) showed a significant effect on Tf-AQF-induced vasorelaxation (Fig. 3I,J).

## DISCUSSION

FTIR fingerprinting of Tf-EE, Tf-HAF and Tf-AQF Fig. S1) suggested the presence of phenolic and carbonyl compounds, evidenced by absorptions related to the stretching of O-H (3000–3500 cm^−1^), C=O (1700 cm^−1^), C=C (1600 cm^−1^) and C-O (1100–1300 cm^−1^) (Pavia et al., 2015). Tf-HEXF showed a profile of compounds of lipidic nature (triacylglycerols and alcohols), evidenced by stretching of O-H (3600–3400 cm^−1^), C-H (strong absorptions at 2800 and 2900 cm^−1^), C=O (1759 cm^−1^) and C-O (1100–1150) (Pavia et al., 2015). UV-Vis fingerprinting of Tf-EE, Tf-HAF and Tf-AQF (Fig. S2) showed two absorptions maxima between 255–265 and 346–358 nm characteristic of flavonol (Mabry et al., 1970; Ferreira et al., 2014).

*Terminalia fagifolia* presents chemical constituents of biological importance, including phenolics and flavonoids (Nunes et al., 2014), which are known for their cardiovascular effects. In species of the same genus, such as *Terminalia arjuna* (Roxb.) Wight & Arn. (Dwivedi, 2007; Kapoor et al., 2014; Manna et al., 2007; Sinha et al., 2008; Singh et al., 2008), *Terminalia chebula* Retz. (Suchalatha and Devi, 2005) and *Terminalia superba* Engler & Diels (Tom et al., 2010), one may find pentacyclic tripertenes and their glycosilated derivates, flavonoids, tannins and other aromatic compounds, which may present cardioprotective activity (Araújo and Chaves, 2005). The thin-layer chromatography performed by Nunes et al. (2014) suggests the identification of compounds similar to (+)-catechin and (−)-epicatechin Tf-EE and Tf-HAF and (−)-epicatechinin Tf-AQF. Some cardioprotective effects such as reductions in systemic blood pressure, atherosclerosis, platelet activation and thrombus formations can be attributed to catechins (Mangels and Mohler, 2017), and the action in which (−)-epicatechin induces vasorelaxation may be dependent on endothelial cells (Moreno-Ulloa et al., 2014).

The results of this research make it clear that Tf-EE presented a vasorelaxant effect that was dependent on concentration and independent of vascular endothelium, E_max_ close to 100% in rings contracted by PHE, with and without vascular endothelium (Table 1). Tf-HEXF or Tf-HAF presented a vasorelaxant effect in a way that was dependent on concentration and independent of endothelium. However, they did not reach 100% of relaxing efficacy (Table 1), since Tf-AQF presented a concentration-dependent vasorelaxant effect that was more potent in rings with vascular endothelium, E_max_ of 100% (Table 1 and Fig. 1).

Tf-AQF induced concentration-dependent vasorelaxation that was more potent in aorta rings with vascular endothelium (Fig. 1). That suggests that metabolites stemming from the vascular endothelium are potentializing the vasorelaxation induced by the Tf-AQF.

Several plants from a number of families induce vasorelaxation in aorta rings through the stimulus of nitric oxide (NO) endothelial release, as in, for example, the species *Schizophyllum commune* (Chen et al., 2014), *Euphorbia humifusa* Willd. (Wang et al., 2013), *Alpiniae zerumbet* (Pers.) B. L. Burtt & R. M. Sm. (Zingiberaceae) (Tao et al., 2013) and *Mansoa hirsuta* D.C. (Campana et al., 2008). This fact reinforces the existence of a common class of compounds widely distributed in the vegetal kingdom, probably the flavonoids, which include a great group of polyphenolic compounds that are also present in the *T. fagifolia* stem bark (Nunes et al., 2014). Those are attributed with the capacity for dilating arteries, stimulating the fast formation of NO, and causing the cyclic guanosine monophosphate (cGMP) levels to rise, as well as opening multiple K^+^ channels through EDHF mediated by endothelial cells (Ajay et al., 2003; Fleming and Busse, 2003).

Once produced by the enzyme endothelial nitric oxide synthase (eNOS), NO activates the sGC in the cytoplasm of the vascular smooth muscle cells and promotes the synthesis of a second intracellular messenger, cGMP, which causes biological effects such as vascular smooth musculature relaxation and inhibition of platelet aggregation. Other mechanisms through which the NO/cGMP pathway induces vasodilatation include inhibition of the inositol triphosphate synthesis, activation of the Na^+^/K^+^-ATPase pump, modulation of the Ca^2+^ and the K^+^ channels, dephosphorylation of the light chain of myosin and reduction of the sensitivity towards vasoconstricting agents (Paolocci et al., 2000; Fleming and Busse, 2003; Adachi et al., 2004).

The activation of the PKG and the consequent phosphorylation of several proteins constitutes a signaling cascade, which may lead to a reduction in the cytoplasmatic concentration of calcium ([Ca^2+^]_i_) and, thus, to vascular relaxation. An increase in the levels of cGMP causes vasodilatation through the activation of kinase proteins dependent on guanosine monophosphate (PKG) (Bredt and Snyder, 1990). In the present study, Tf-AQF presents a relaxing effect that is more potent in aorta rings with vascular endothelium, suggesting that the participation of metabolites from the endothelium is potentializing the vascular relaxation.

The EDHFs, primarily NO, may generate an increase in the membrane potential in certain tissues, which develops a hyperpolarization dependent on the vascular endothelium (Zygmunt et al., 1994; Petersson et al., 1995). The E_max_ induced by Tf-AQF in the aorta rings with vascular endothelium was 103.83±2.43%, which is typical of a cellular hyperpolarization that involves the action of EDHFs (Fig. 1).

The experiments on aorta rings isolated from rats showed that Tf-AQF promotes vasorelaxation dependent on the NO/cGMP pathway, the curve was shifted to the right in the presence of an NO synthase inhibitor (L-NAME 300 μM, Fig. 2A), or a guanyllyl cyclase inhibitor (ODQ 10 μM, Fig. 2C); or an extra and intracellular NO remover (PTIO 300 μM, Fig. 2E).

It has been confirmed that the inhibition of the synthase NO enzyme by the use of L-NAME significantly moved the relaxation curve to the right, with a reduction in the pharmacological efficacy and potency of the vasorelaxant response of the rings promoted by Tf-AQF (control: pD_2_=1.98±0.02; L-NAME: pD_2_=2.21±0.04*/control: E_max_=103.83±2.43; L-NAME: E_max_=80.26±8.93*) (Fig. 2A). The presence of nitric oxide in the vascular smooth muscle activates the sGC that act over the guanosine triphosphate, converting it to cGMP, which is important in the regulation of vascular tonus. The inhibition of the soluble guanylyl cyclase enzyme can be performed with the use of ODQ (Yeh et al., 2005; Hipólito et al., 2009), and when the aorta rings were pre-incubated with ODQ, the curve of relaxation induced by Tf-AQF moved significantly to the right with attenuation of the relaxing efficacy, in the presence of that inhibitor (Fig. 2B).

The NO scanning of both the intra- and extracellular media can be performed with the use of PTIO (Dantas et al., 2014); in the presence of that pharmacological tool, the vasorelaxation curves induced by Tf-AQF shifted significantly to the right in the presence of PTIO. The results suggest that vascular relaxation induced by Tf-AQF involves NO/sGC/cGMP signaling pathways, and when pharmacological tools that comprise the action of NO/sGC/cGMP were used, the vasorelaxation curve induced by the fraction presented a significant reduction in pharmacological potency (Fig. 2E).

NO induces the relaxation of the vascular smooth musculature, mainly through cellular processes: activation of the sGC with the synthesis of cGMP (Moncada et al., 1989) and direct activation of the K^+^ channels (Villar et al., 2006), a mechanism that shows close relation to natural products, such as plant extracts (Mcneill and Jurgens, 2006; Schmitt and Dirsch, 2009). Based on these data, we propose to investigate the participation of K^+^ channels in the vasorelaxant effect induced by Tf-AQF in aorta rings isolated from rats.

K^+^ channels contribute to the regulation of membrane potential, as well as the contractility of both the musculature and vascular tonus (Jackson, 2005; Ko et al., 2008). Relaxing factors derived from the endothelium, such as NO, induce vasorelaxation by activating K^+^ channels, closing Ca^2+^channels in the smooth muscle (Nelson and Quayle, 1995). Thus, in order to verify the participation of K^+^ channels in the vasorelaxant effect induced by Tf-AQF, the thoracic aorta rings were incubated with the following pharmacological tools for 30 min: TEA (3 mM), non-selective blocker of K^+^ channels; GLIB (10 μM), a blocker of K^+^ channels sensitive to ATP (Mcneish et al., 2006); 4-AP (1 mM), voltage-dependent blocker of K^+^ channels (Chen et al., 2009); APM (0.1 μM), selective blocker of K^+^ channels activated by low-conductance Ca^2+^ (Wang et al., 2013); ChTX (0.1 μM), selective blocker of K^+^ channels activated by intermediate-conductance Ca^2+^ (Wang et al., 2013).

NO is described as an activator of K^+^ channels in the membrane of the smooth muscle cells and indirectly through PKG (Archer et al., 1994; Baranowska et al., 2007). The increased conductance of the K^+^ ions hyperpolarizes the cells and reduces Ca^2+^ ion influx through voltage-operated channels, present in the cellular membrane (Nelson and Quayle, 1995; Jackson, 2005), thus promoting the relaxation of the vascular smooth musculature.

Our results show that the relaxing efficacy of Tf-AQF was reduced in the presence of TEA (non-selective blocker of K^+^ channels), with the curve being shifted to the right, and attenuation of the pharmacological potency (Fig. 3A). Based on these results, one can suggest that the K^+^ channels are involved in the relaxing mechanism of the rat aorta rings induced by Tf-AQF.

In light of these data that show evidence of the participation of K^+^ channels in the vasorelaxant mechanism of Tf-AQF, different pharmacological tools have been used in order to identify which types of channels are involved in the vasorelaxant response of the substance researched.

The activation of K_ATP_ channels can stem from the release of endothelium-derived factors, such as NO. NO and cGMP may promote a hyperpolarization and relaxation of the smooth muscular cells by means of the opening of K_ATP_ channels (Feleder and Adler-Graschinsy, 1997; Yeh et al., 2005). Therefore, nitric oxide donor substances have been shown as activators of the K_ATP_ independently on cGMP (Murphy and Brayden, 1995), and by an independent mechanism of cGMP in the rat mesenteric artery (Weidelt et al., 1997).

The vasorelaxant response induced by Tf-AQF was not altered in the presence of the selective blocker of K^+^ channels sensitive to ATP and GLIB (Tep-Areenan et al., 2003; Ko et al., 2008). In the presence of 4-AP, an inhibitor of voltage-dependent K^+^ channels (K_V_) (Ko et al., 2008), the curve of vascular relaxation induced by Tf-AQF (Fig. 3E) was shifted to the right, with a reduction of the pharmacological efficacy, highlighting the participation of K_V_ channels in the vasorelaxant effect induced by Tf-AQF. Hence, it has been suggested that K_V_ channels are involved in the relaxant response induced by Tf-AQF.

In order to verify the role of Ca^2+^-activated K^+^ channels in the vasorelaxation induced by Tf-AQF in aorta rings, specific blockers were used according to the subtype of K^+^.

Endothelial K^+^ pharmacological potency has been widely implicated in endothelium-dependent vasodilatation, mainly the low-conductance Ca^2+^-activated K^+^ channels (SK_Ca_) and the intermediate-conductance Ca^2+^-activated K^+^ channels (IK_Ca_). Initially, it has been considered that the hyperpolarization of the endothelial cells, through the opening of K^+^ channels, increases the production of EDHF, such as NO and PGI_2_, and triggers a vasodilatating response (Coleman et al., 2004; Köhler et al., 2010).

In the presence of apamin (APM) or charybdotoxin (ChTX), blockers of the SK_Ca_ or BK_Ca_ channels, respectively, the vasorelaxant effect induced by Tf-AQF did not present a significant difference between the pD_2_ values (Fig. 3G,I). Hence, the SK_Ca_ and BK_Ca_ channels seem not to be involved in the relaxing response of Tf-AQF.

The existence of EDHF was proposed based on the observations that a substance released from the endothelium causes hyperpolarization of the cells of the vascular smooth muscle (Chen et al., 1988; Vanhoutte, 1997). Besides, recent studies have supplied strong evidence of the fact that EDHF is predominant in endothelium-dependent vascular relaxation (Huang et al., 2004).

In conclusion, our data show that Tf-EE has a non-endothelium-dependent vasorelaxant effect in aorta rings pre-contracted with PHE; additional studies are necessary in order to understand the vasorelaxant mechanism. Tf-AQF is capable of leading to endothelium-dependent vasorelaxation in aorta rings isolated from rats, and the mechanism of endothelium-dependent vasodilatation is entirely related to the activation of the eNOS/NO/cGMP system and as well as the participation of K_V_. Further studies must be conducted for the investigation of the efficacy and safety of the substance *in vivo*, in the acute form and in the chronic use of preparations obtained from *T. fagifolia*.

## MATERIALS AND METHODS

### Plant material

The stem bark of *T.* *fagifolia* Mart. & Zucc. (Combretaceae) was collected in November 2006 from the ‘Bambu’ community, Timon-MA, Brazil. A voucher specimen (TEPB number 21.691) was deposited in the Graziela Barroso Herbarium at the Federal University of Piauí, Teresina, state of Piauí, Brazil. The extract and the fractions of *T. fagifolia* were obtained by Professors Paulo Humberto Moreira Nunes and Mariana Helena Chaves.

### Extraction of the extract and fractions

The vegetal material was dried in the shade at 40°C, and the powder from the stem bark was continuously extracted with ethanol 99.6%. After the filtration, the solvent was removed under vacuum at 50°C and the concentrate was lyophilized to yield the ethanolic extract of the *T. fagifolia* stem bark (Tf-EE), which was stored under refrigeration until the moment of use.

In order to obtain its partition fractions, Tf-EE was dissolved in a solution of methanol/distilled water (1:2) and extracted with ethyl acetate. Following this, the ethyl acetate phase was concentrated and dissolved in a solution of methanol/distilled water (9:1) and extracted with hexane. The phases obtained were concentrated with solvent removal and resulted in the aqueous partition fraction (Tf-AQF), hydroethanolic fraction (Tf-HAF) and hexanic fraction (Tf-HEXF).

### FTIR and UV-Vis fingerprinting

FTIR and UV-Vis spectra were recorded on a Spectrum 100 spectrometer (PerkinElmer) using KBr pellets and on a Lambda 25 spectrophotometer (PerkinElmer), in methanol at a concentration of 0.05 mg/ml.

### Chemicals and drugs

Phenylephrine (PHE), acetylcholine chloride (ACh), N^G^-nitro-L-arginine methyl ester (L-NAME), 1H-[1,2,4]Oxadiazole[4,3-a]quinoxalin-1-one (ODQ), 2-Phenyl-4,4,5,5-tetramethylimidazoline-1-oxyl 3-oxide (PTIO), glibenclamide (GLIB), 4-aminopyridine (4-AP), tetraethylammonium chloride (TEA), apamin (APM) and charybdotoxin (ChTX) were obtained from Sigma-Aldrich (USA).

ODQ and GLIB were dissolved in dimethyl sulfoxide (DMSO, 0.1%); PTIO in ethanol; and APM in 0.05 M acetic acid. The other drugs and reagents were dissolved in distilled water.

### Animals

Male Wistar rats weighing 250–300 g each, were obtained from the animal facility of the Medicinal Plants Research Center - UFPI, maintained under controlled temperature (24±1°C) and light/dark cycle and allowed to free access to food and water. The project was approved by the Ethics Committee for Animal Experiments -UFPI, with the report number 008/12.

### Tissue preparation

All rats were euthanized by an overdose with Thiopental sodium in intraperitoneal administration (100 mg/kg, i.p.). The aortic rings, measuring about 3–4 mm, were obtained from the thoracic aorta and were freed from the surrounding connective tissues. To obtain isometric responses, the rings were individually suspended on stainless steel rods in organ baths (6 ml) containing normal Krebs solution at 37°C with the following composition (mM): NaCl (118.0), NaHCO_3_ (25.0), KCl (4.6), MgSO_4_ (5.7), KH_2_PO_4_ (1.1), CaCl_2_ (2.5) and glucose (11.0), at 37°C and pH 7.4. After 60 min of equilibration period, the bath solution was replaced every 15 min to prevent metabolite interference (Altura and Altura, 1970). During this period, the aortic rings were kept under a resting tension of 1 g. In the meantime, the nutrient solution was renewed every 15 min to prevent metabolite interference (Altura and Altura, 1970). In some of the rings, usual care was taken to avoid abrasion of the inner surface and maintain the integrity of the endothelial layer. In the remaining aortic rings, endothelial cells were removed by gently rubbing the inner surface with moistened cotton swabs. After the initial stabilization period, a contraction was induced with PHE (1 µM) and when the tonic phase was reached (12–15 min), ACh (1 µM) was added to all preparations to verify endothelium integrity (Furchgott and Zawadzki, 1980). The vascular endothelium was considered intact when the aortic rings showed more than 50% of relaxation (Ajay et al., 2003). Endothelium removal was confirmed by the absence of relaxation after ACh was added to the bath, or when the relaxation was lower than 10%, which meant these rings were considered free of functional endothelium.

### Effects of Tf-EE and its partition fractions on the contraction of rat thoracic aortic rings with intact or denuded endothelium induced by PHE

To assess whether the extract and fractions of *T. fagifolia* had relaxing effect, the rings were pre-contracted with PHE (1 μM), and after the contraction reached the tonic phase, Tf-EE, Tf-HEXF, Tf-HAF or Tf-AQF were cumulatively added (0.1–750 μg/ml), in both endothelia with and without aortic rings.

### Effect of the Tf-AQF on PHE-induced tonic contractions in endothelium-intact rat aortic rings in the presence of L-NAME, ODQ or PTIO

Aorta was obtained as described in the previous section. Before inducing the second PHE contraction in rings with functional endothelium, the following agents were added in different preparations: L-NAME (300 µM for 30 min), ODQ (10 µM for 20 min) or PTIO (300 µM for 30 min). In the tonic component of the second PHE-induced contraction in the presence of each blocker, Tf-AQF (0.1–750 μg/ml) was cumulatively added to the bath in different preparations.

### Effect of Tf-AQF on PHE-induced tonic contractions in endothelium-intact rat aortic rings in the presence of GLIB, 4-AP, TEA, APM or ChTX

Aorta was obtained as described in the previous section. Before inducing the second PHE-induced contraction in rings with intact endothelium, TEA (3 mM for 30 min), GLIB (10 μM for 30 min), 4-AP (1 mM for 30 min), APM (1 μM for 30 min) or ChTX (0.1 μM for 30 min) was added in different preparations. In the tonic component of the second PHE-induced contraction in the presence of blockers, Tf-AQF (0.1–750 μg/ml) was cumulatively added to the bath in different preparations. The relaxation was expressed as previously described.

### Statistical analysis

Data were shown as mean±s.e.m. and comparison between two values was assessed by unpaired Student's *t*-test, considering statistical significance at a value of *P*<0.05. The pD_2_ values (defined as cologarithm of the concentration of extract that induced 50% of the maximal relaxation) and E_max_ values (values of maximal relaxation) were determined by fitting the original dose-response curves using the GraphPad Prism software (version 5.0).

## Supplementary Material

Supplementary information
