# Supplementary material for: Terminalia fagifolia Mart. & Zucc. elicits vasorelaxation of rat thoracic aorta through nitric oxide and K+ channels dependent mechanism
Source: Biol Open. 2019 Jan 25;8(2):bio035238. doi: 10.1242/bio.035238 (PMC6398462; doi:10.1242/bio.035238)
Supplement: Supplementary information [file biolopen-8-035238-s1.pdf]

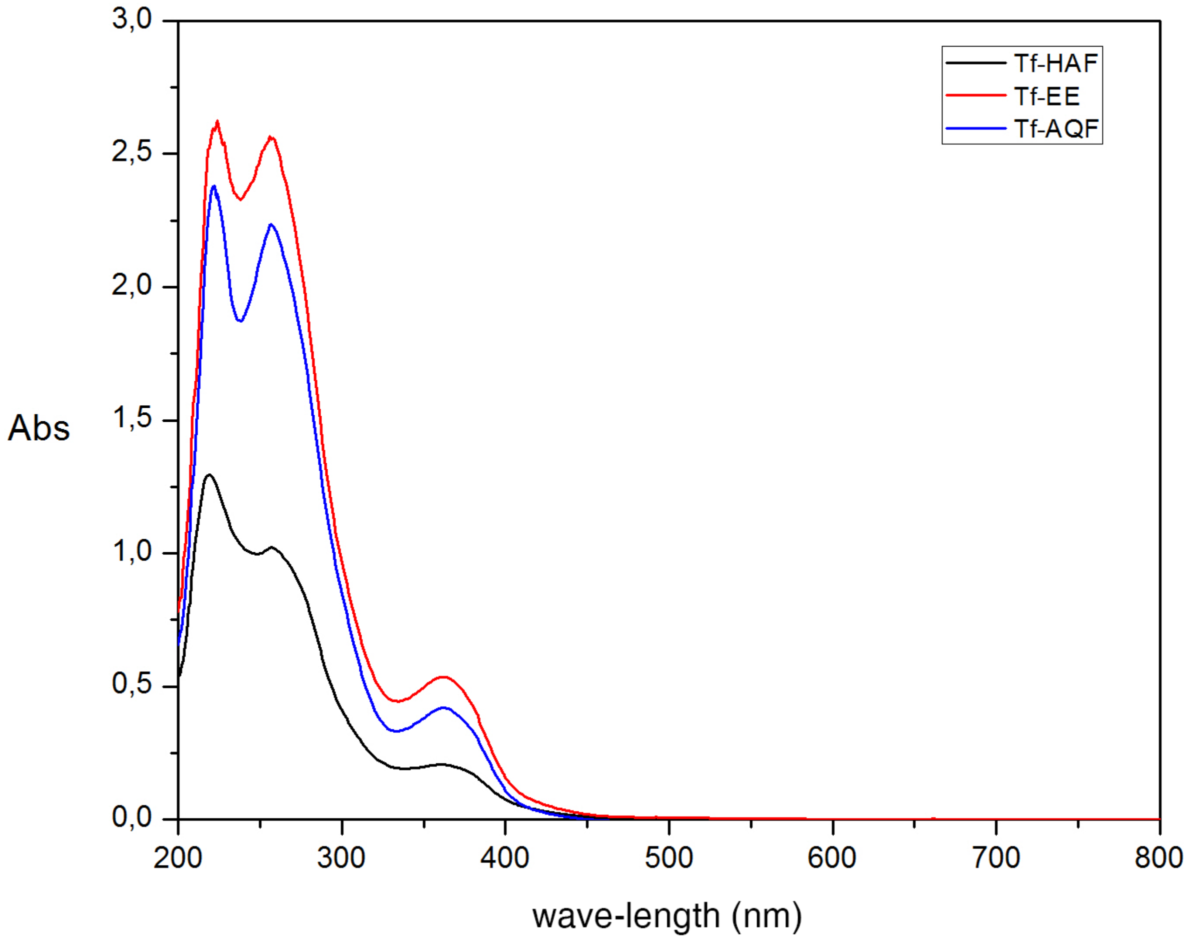

Figure S1. FTIR fingerprinting of the extract and fractions of *T. fagifolia* (Tf-EE, Tf-HEXF Tf-HAF and Tf-AQF).

Biology Open • Supplementary information

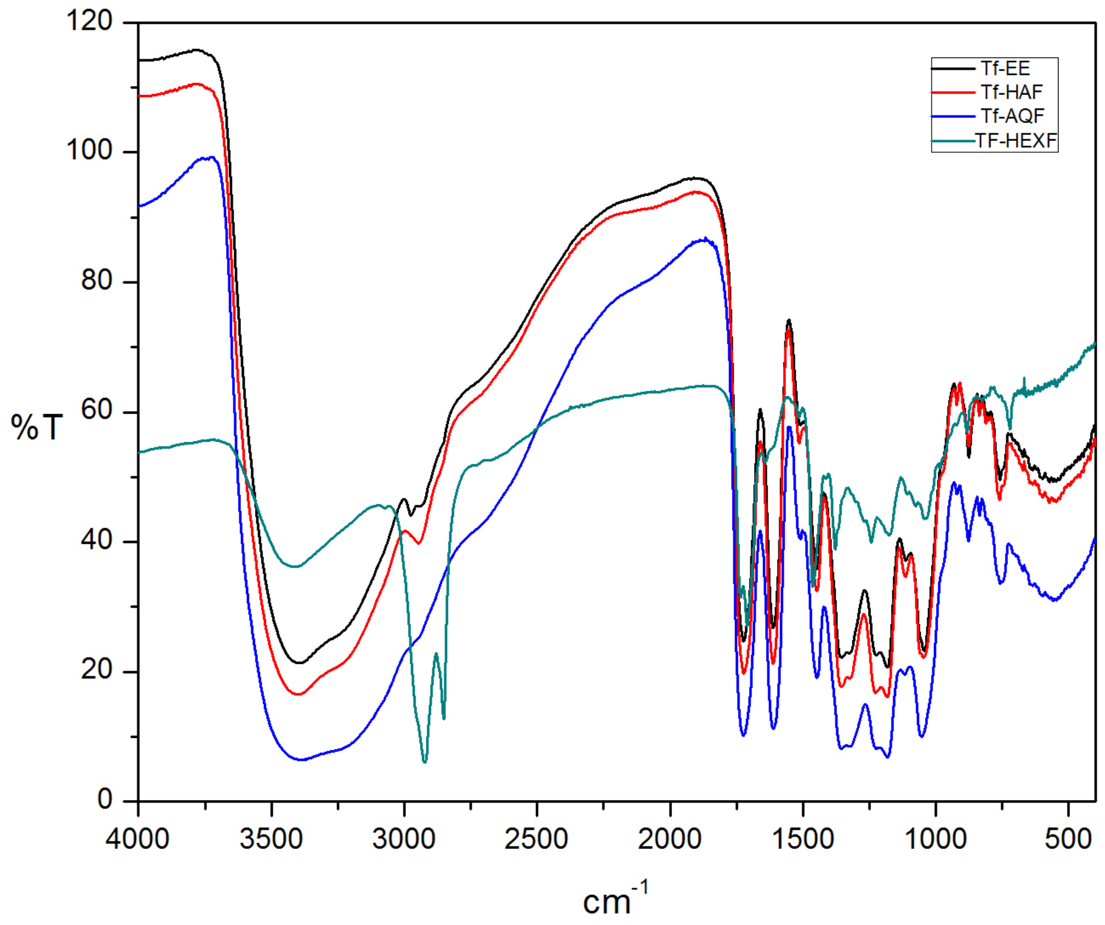

Figure S2. UV-Vis fingerprinting of the extract and fractions of *T. fagifolia* (Tf-EE, Tf-HAF and Tf-AQF).

Biology Open • Supplementary information
